# Does the Use of Peripheral Immune-Related Markers Indicate Whether to Administer Pazopanib, Trabectedin, or Eribulin to Advanced Soft Tissue Sarcoma Patients?

**DOI:** 10.3390/jcm10214972

**Published:** 2021-10-26

**Authors:** Eijiro Shimada, Makoto Endo, Yoshihiro Matsumoto, Kenji Tsuchihashi, Mamoru Ito, Hitoshi Kusaba, Akira Nabeshima, Tomoya Nawata, Akira Maekawa, Tomoya Matsunobu, Nokitaka Setsu, Toshifumi Fujiwara, Keiichiro Iida, Makoto Nakagawa, Takeshi Hirose, Masaya Kanahori, Ryunosuke Oyama, Taichi Isobe, Hiroshi Ariyama, Kenichi Kohashi, Hidetaka Yamamoto, Yoshinao Oda, Yukihide Iwamoto, Koichi Akashi, Eishi Baba, Yasuharu Nakashima

**Affiliations:** 1 Department of Orthopaedic Surgery, Graduate School of Medical Sciences, Kyushu University, 3-1-1, Maidashi, Higashi-ku, Fukuoka 812-8582, Japan; e.shimada1987@gmail.com (E.S.); ymatsu@ortho.med.kyushu-u.ac.jp (Y.M.); nabeshi.com@gmail.com (A.N.); sets0rockandsnow@gmail.com (N.S.); to-fu-a@ortho.med.kyushu-u.ac.jp (T.F.); iida-k16@ortho.med.kyushu-u.ac.jp (K.I.); nakagawam59@gmail.com (M.N.); thedays_goby@yahoo.co.jp (T.H.); k.masaya128@gmail.com (M.K.); ryunosuke.o.tz@gmail.com (R.O.); yasunaka@ortho.med.kyushu-u.ac.jp (Y.N.); 2 Department of Hematology, Oncology and Cardiovascular Medicine, Kyushu University Hospital, 3-1-1, Maidashi, Higashi-ku, Fukuoka 812-8582, Japan; kenji.tsuchihashi@gmail.com (K.T.); ito.mamoru.632@m.kyushu-u.ac.jp (M.I.); hkusaba@intmed1.med.kyushu-u.ac.jp (H.K.); hariyama@intmed1.med.kyushu-u.ac.jp (H.A.); akashi@intmed1.med.kyushu-u.ac.jp (K.A.); 3 Department of Orthopaedic Surgery, Kyushu Rosai Hospital, 1-1, Sonekita, Kokuraminami-ku, Kitakyushu, Fukuoka 800-0296, Japan; e.21.6611bd@gmail.com (T.N.); maekawarugby@gmail.com (A.M.); matsunob@ortho.med.kyushu-u.ac.jp (T.M.); yiwamoto@ortho.med.kyushu-u.ac.jp (Y.I.); 4 Department of Oncology and Social Medicine, Graduate School of Medical Sciences, Kyushu University, 3-1-1, Maidashi, Higashi-ku, Fukuoka 812-8582, Japan; tisobe@intmed1.med.kyushu-u.ac.jp (T.I.); e-baba@intmed1.med.kyushu-u.ac.jp (E.B.); 5 Department of Anatomic Pathology, Graduate School of Medical Sciences, Kyushu University, 3-1-1, Maidashi, Higashi-ku, Fukuoka 812-8582, Japan; kohas@surgpath.med.kyushu-u.ac.jp (K.K.); hidetaka@surgpath.med.kyushu-u.ac.jp (H.Y.); oda.yoshinao.389@m.kyushu-u.ac.jp (Y.O.)

**Keywords:** soft tissue sarcoma, chemotherapy, pazopanib, trabectedin, eribulin, prognosis, biomarker, peripheral immune-related marker, neutrophil to lymphocyte ratio, platelet to lymphocyte ratio

## Abstract

Pazopanib, trabectedin, and eribulin are administered for the treatment of soft tissue sarcomas (STSs); however, there is little consensus on which agent should be preferentially used in a clinical setting. This study assessed whether peripheral immune-related markers served as a useful reference when selecting pazopanib, trabectedin, or eribulin. This study included 63 patients who were administered pazopanib, trabectedin, or eribulin for advanced STSs between March 2015 and December 2020. Patients were divided into three groups based on the first drug administered among these three drugs. Differences in overall survival (OS) or progression-free survival (PFS) among the three groups were analyzed. OS showed no significant differences among the drugs administered first. For patients with low neutrophil-to-lymphocyte ratio (NLR), the OS of patients administered pazopanib as the first choice was shorter than the others (hazard ratio [HR] = 9.53, 95% confidence interval [CI] = 1.94–18.13, *p* = 0.0018). In the low platelet-to-lymphocyte ratio (PLR) subgroup, the OS of the patients administered eribulin for the first choice was longer than that of the others (HR = 0.32, 95%CI = 0.10–0.98, *p* = 0.046). Therefore, NLR and PLR might be used as prognostic indicators to dictate whether STS patients receive pazopanib, trabectedin, or eribulin.

## 1. Introduction

Soft tissue sarcomas (STSs) are rare malignant tumors with a wide variety of histological and biological behaviors. The prognoses of advanced STSs with unresectable local recurrence and/or metastasis remain poor with a median overall survival (OS) of approximately 7.7–38.9 months [1]. In the past, only a limited number of drugs such as doxorubicin, ifosfamide, gemcitabine, and docetaxel were administered as systemic therapies for STSs [2,3]. However, three different types of agents, pazopanib, trabectedin, and eribulin, have been approved and used for STSs in the early years of the 21st century [4,5,6]. Because there are no head-to-head comparative trials among these three agents or good biomarkers indicating the effectiveness of each treatment, a consensus on which agent should be preferentially used has not been reached.

Peripheral immune-related markers such as absolute lymphocyte count (ALC), neutrophil-to-lymphocyte ratio (NLR), platelet-to-lymphocyte ratio (PLR), and lymphocyte-to-monocyte ratio (LMR) have been attracting attention as factors that predict cancer prognosis [7,8,9,10,11]. The first report on immune-related markers was that the NLR correlated with the prognoses of intensive care unit patients [12]. Later, it was reported that peripheral immune-related markers correlated with prognosis in colorectal cancer [7], and this correlation has been reported in several other cancers [8,9,10,11]. In STSs, there are also a couple of reports that show that immune-related markers correlate with prognosis [13,14,15,16]; however, to the best of our knowledge, there are no reports that show whether peripheral immune-related markers serve as a useful reference for selecting one of the candidate agents for STSs.

The purpose of this study was to verify whether peripheral immune-related markers can predict the prognosis of patients with STSs who were treated with pazopanib, trabectedin, and/or eribulin.

## 2. Materials and Methods

### 2.1. Patients

This study was a retrospective, two-facility joint study at Kyushu University Hospital and Kyushu Rosai Hospital. In this study, patients who had been administered pazopanib, trabectedin, and/or eribulin for advanced STS between March 2015 and December 2020 were included. Patients whose detailed medical records were unavailable were excluded. The patients were then divided into three groups based on the first drug administered among the three drugs. Pazopanib (800 mg) was administered orally once daily. Trabectedin (1.2 mg/m^2^) was administered intravenously on day 1 of every 21-day cycle. Eribulin mesylate (1.4 mg/m^2^) was administered intravenously on days 1 and 8 of every 21-day cycle. The choice of agent, dosage reduction, and intervals of administration were left to the physician’s discretion by reference to the usage guide of each drug. The requirement for informed consent was waived because the data were collected anonymously. Study approval was obtained from the Kyushu University Institutional Review Board (approval No. 26–224). This study was conducted in accordance with the Declaration of Helsinki.

### 2.2. Methods

ALC, NLR, PLR, and LMR were calculated from blood samples collected within a week before administration of the first drug. The cutoff values were set as follows: 1500 for ALC, 3 for NLR, 200 for PLR, and 3 for LMR based on previous studies [17,18,19]. The following patient demographics were obtained from medical records: age, sex, location of the primary tumor, surgical resection of the primary tumor, histological diagnosis, Eastern Cooperative Oncology Group performance status (ECOG PS) at the start of drug administration, number of previous chemotherapies, previous administration of doxorubicin or ifosfamide, number of following chemotherapies, and history of radiotherapy or carbon-ion radiotherapy. Demographic factors were stratified as follows: age <65 or ≥65 years; location of the primary tumor on the extremities or elsewhere; histological classification as L-sarcoma (leiomyosarcoma and liposarcoma) or other; ECOG PS of 0 or ≥1; 0–1 or ≥2 previous chemotherapies. Hematological adverse events during the first course of each drug administration were evaluated according to the National Cancer Institute Common Terminology Criteria for Adverse Events v5.0.

### 2.3. Statistical Analysis

Fisher’s exact test was used to compare the proportions of categorical variables among groups. OS was defined as the time from the date of drug administration to the date of death. OS was censored at the last date when the patient was known to be alive. Progression-free survival (PFS) was defined as the time from the date of the applicable drug administration to the date of radiological disease progression or death, whichever occurred first. PFS was censored at the last date when the patients ceased the applicable drug administration from any cause without disease progression. OS and PFS were estimated using the Kaplan–Meier method and compared with log-rank test. Univariate analyses for factors were performed using the Cox proportional hazards model. The threshold for significance was set at *p* < 0.05. All statistical analyses were performed using JMP^®^ 14 (SAS Institute Inc., Cary, NC, USA).

## 3. Results

### 3.1. Patients and Group Demographics

**Figure 1:**
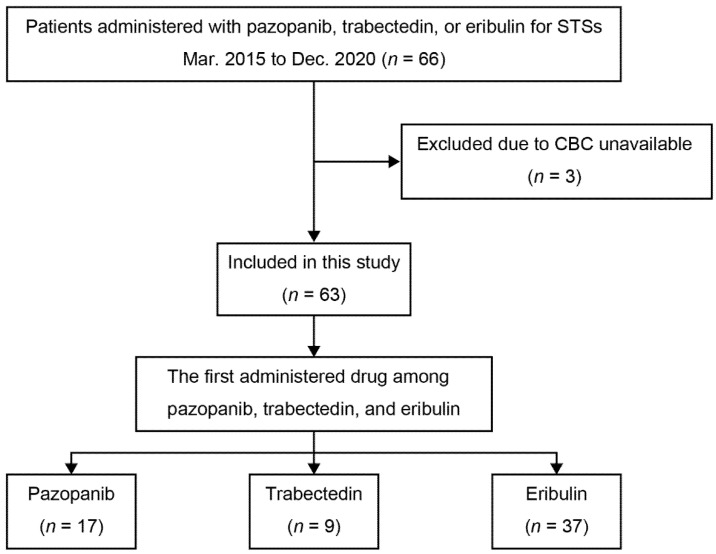
Flowchart of the study group selection process. STSs, soft tissue sarcomas. CBC, complete blood counts.

In total, 63 patients were included in this study whereas three patients were excluded because their complete blood counts were not available (Figure 1). The median age was 66 years, and the median follow-up was 510 days. Eribulin was the first drug administered to 37 patients, pazopanib was the first for 17 patients, and trabectedin was the first for nine patients. Some overlapping in the administration of eribulin, pazopanib, and trabectedin were observed (Table S1). There were no statistical associations between the drugs first administered and demographic variables such as age and the number of previous chemotherapies, except for histology. L–sarcoma was identified for 64.9% of patients first given eribulin, 17.7% for patients first given pazopanib, and 66.7% for patients first given trabectedin (*p* = 0.0036) (Table 1, Table S2).

**Figure 2:**
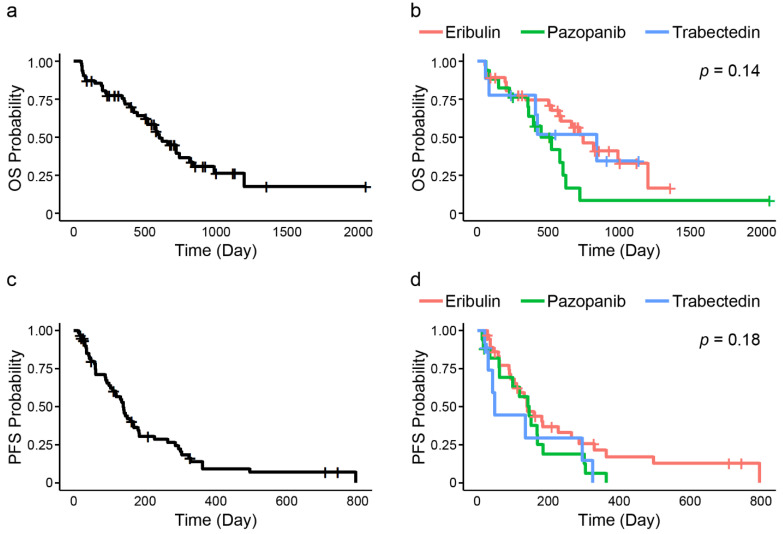
OS and PFS for advanced STSs patients. (**a**) OS for all STSs patients; (**b**) Comparison of OS among the first administered drugs. There were no differences among the first administered drugs; (**c**) PFS for all STSs patients; (**d**) Comparison of PFS among the first administered drugs. There were no differences among the first administered drugs.

The median OS for all patients was 602 days (95% confidence interval [95% CI] = 519–838) (Figure 2a). The choice of the first administered drug did not significantly influence OS (median: 744 days [95% CI = 519–1198] for eribulin, 520 days [95% CI = 223–602] for pazopanib, and 838 days [95% CI = 56–not reached (NR)] for trabectedin; *p* = 0.14) (Figure 2b). Univariate analysis of all the included cases showed that low NLR (HR = 0.50; 95% CI = 0.26–0.96; *p* = 0.037) and low LMR (HR = 2.24; 95% CI = 1.17–4.31; *p* = 0.015) were factors associated with OS (Table 2).

The median PFS for all patients was 139 days (95% CI, 103–169) (Figure 2c). PFS was also not significantly determined by the choice of the first administered drug (median: 139 days [95% CI = 103–227] for eribulin, 145 days [95% CI = 60–169] for pazopanib, and 48 days [95% CI = 20–295] for trabectedin; *p* = 0.18) (Figure 2d). Univariate analysis showed that none of the variables, including peripheral immune-related markers, were associated with PFS (Table S3).

### 3.2. Subgroup Analysis of Peripheral Immune-Related Markers for Survival

In each subgroup divided by peripheral immune-related markers into high and low subgroups, significant differences in OS were identified among the first administered drugs in the low NLR subgroup (median: 816 days [95% CI = 587–1198] for eribulin, 520 days [95% CI = 148–602] for pazopanib, and NR for trabectedin; *p* = 0.0019) and in the low PLR subgroup (median: 1198 days [95% CI = 587–1198] for eribulin, 579 days [95% CI = 148–719] for pazopanib, and 838 days [95% CI = 408–NR] for trabectedin; *p* = 0.024) although there were no significant differences in OS in the other subgroups (Figure S1).

**Figure 3:**
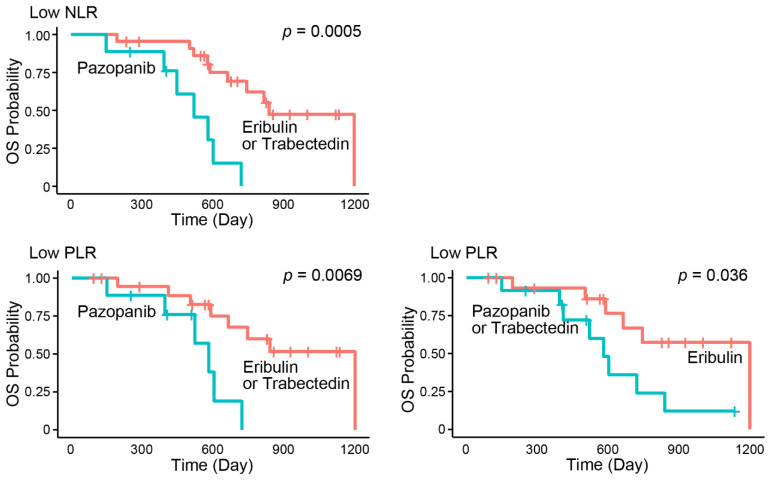
Subgroup analysis of OS among the first administered drugs. In the low NLR and the low PLR subgroup, pazopanib was associated with shorter OS than the others. In the low PLR subgroup, eribulin was associated with longer OS than the others. NLR, neutrophil–to–lymphocyte ratio. PLR, platelet–to–lymphocyte ratio.

In the low NLR subgroup, univariate analysis and the Kaplan–Meier method showed that only pazopanib associated with shorter OS (HR = 5.93; 95% CI = 1.94–18.13; *p* = 0.0018, median = 520 days [95% CI = 148–602] for pazopanib; 838 days [95% CI = 587–1198] for other treatments; *p* = 0.0005). In the low PLR subgroup, univariate analysis and Kaplan–Meier method showed that eribulin was a factor associated with longer OS (HR = 0.32; 95% CI = 0.10–0.98; *p* = 0.046, median = 1198 days [95% CI = 587–1198] for eribulin; 579 days [95% CI = 394–838] for other treatments; *p* = 0.036), and pazopanib was the factor associated with shorter OS (HR = 4.71; 95% CI = 1.38–16.04; *p* = 0.013, median = 579 days [95% CI = 148–719] for pazopanib; 1198 days [95% CI = 587–1198] for other treatments; *p* = 0.0069) (Table 3, Figure 3).

PFS was not significantly different based on the first administered drugs in each subgroup (Figure S2).

Hematological adverse events during the first course of each drug administration showed significant differences in the incidence of lymphopenia (Table S4). In the eribulin group, the low ALC subgroup and the high PLR subgroup were more likely to suffer from lymphopenia (*p* = 0.049, *p* = 0.014, respectively). Additionally, in the pazopanib group, the high NLR subgroup and the high PLR subgroup were more likely to suffer from lymphopenia (*p* = 0.032, *p* = 0.032, respectively).

## 4. Discussion

The ideal way to administer pazopanib, trabectedin, and eribulin for advanced STSs, including their order, remains unclear, which calls for a useful reference for drug selection. Peripheral immune-related markers such as ALC, NLR, PLR, and LMR are easily available because they can be calculated using only complete blood counts, which are examined in routine clinical practice. There are a couple of reports about the relationship between peripheral immune-related markers and several types of cancer including STS. Among them, there is a report about the effectiveness of peripheral immune-related markers as indicators for the use of eribulin for breast cancer. That study showed that high ALC indicated a better prognosis in patients treated with eribulin although ALC did not have any correlation with prognosis in patients who were treated at their physicians’ discretion [17]. As for STS, there are a few reports on the correlation between peripheral immune-related markers and prognosis. It has been reported that the combination of NLR and PLR can predict survival after resection of STS [13], and low NLR and high LMR are correlated with better prognosis in patients with synovial sarcoma [14]. In addition, preoperative low NLR and high LMR were prognostic markers for predicting better clinical outcomes in sarcoma patients after surgery [15], and a low NLR can predict better, durable clinical benefits and longer PFS in STS patients treated with eribulin [16]. Although there are a few studies about peripheral immune-related markers in STS, none of them have validated that peripheral immune-related markers can be a reference for selecting drugs. This study is therefore the first to show the usefulness of peripheral immune-related markers in the selection of antitumor drugs for STSs.

One of the possible underlying mechanisms of peripheral immune-related markers as useful references is the effect of the drugs on the tumor microenvironment. It was reported that eribulin affected intratumoral vascular remodeling that resulted in improved drug delivery and immune cell trafficking [20]. This indicated that the antitumor effect of eribulin was potentially enhanced by a high lymphocyte ratio in the tumor microenvironment. Trabectedin is known to decrease the number of tumor-associated macrophages, which help the tumor to escape from the immune system [21,22]. This indicates that trabectedin creates a tumor environment in which lymphocytes can work more effectively. In summary, patients with low NLR or low PLR, or in other words a high lymphocyte ratio in blood, benefit more from the immune-related effects of eribulin and trabectedin, and this leads to improved OS.

The next concern is why only NLR and PLR served as references for drug selection while ALC and LMR did not. This may be because peripheral immune-related markers reflect not only the anti-oncogenicity status of the immune system but also the pro-oncogenicity status. Because the number of lymphocytes may potentially reflect the immune microenvironment within the tumor, ALC is considered an effective biomarker that reflects anti-oncogenicity [17]. On the contrary, neutrophils reflect a state of host inflammation and can promote oncogenic processes including tumor growth [23]. Platelets also contribute to cancer progression by promoting critical processes. For example, platelets inhibit the activation of T-helper cell 17 responses that enhance tumor proliferation [24]. While ALC reflects only anti-oncogenicity, NLR and PLR reflect the balance of pro- and anti-oncogenicity, which may contribute to the differences in the results. There are several reports supporting this hypothesis that conclude that NLR and PLR are useful biomarkers while ALC is not [15,16].

This study has some limitations. First, the number of patients analyzed here was not sufficient to determine the roles of all peripheral immune-related markers in STSs. We found that pazopanib was associated with shorter OS in the low NLR and low PLR subgroups, and eribulin was associated with longer OS in the low PLR subgroup. However, there were no significant findings in the high NLR, high PLR, or high or low ALC or LMR subgroups. There is a possibility of an inadequate sample size for these subgroup analyses. Further validation, including multivariate analysis, with a larger number of patients is necessary in the future. Second, because this study is retrospective in nature, it may contain a variety of biases. Especially, the choice of drugs including the dosages and number of courses depended on the physician’s choice. Although pazopanib, trabectedin, and eribulin can be administered to all STS subtypes in Japan, the administration of pazopanib for liposarcoma is likely to be avoided because its phase III clinical trial did not include patients with liposarcoma [4]. In addition, eribulin was preferred for treatments because it was considered to have relatively mild adverse effects [25]. Such considerations affect the physician’s choice and result in a difference among the groups. Generally speaking, the heterogeneity of soft tissue sarcomas and the numerous subtypes with different levels of chemosensitivity make clinical research on chemotherapy challenging. Third, there were also possibilities that OS was affected by overlapping between the drugs. Considering these limitations, careful interpretation of the results might be needed. To overcome these biases in the patient’s background, we are conducting a randomized, controlled prospective study to compare the effects of pazopanib, trabectedin, and eribulin.

In conclusion, this study demonstrated that eribulin or trabectedin for patients with low NLR and eribulin for patients with low PLR were associated with longer OS compared to the other drugs, whereas the OS differences were not statistically significant in the analysis of the whole patient cohort without grouping by peripheral immune-related markers. This study generated an interesting hypothesis on the usefulness of peripheral immune-related markers, which warranted further study in a larger, prospective trial. When selecting either pazopanib, trabectedin, or eribulin for advanced STSs, NLR, and PLR served as useful references that should be considered clinically.

## Figures and Tables

**Table 1:** Baseline demographics of the study patients (n = 63).

		Eribulin (*n* = 37)	Pazopanib (*n* = 17)	Trabectedin (*n* = 9)	*p*–Value
Age	≥65	19 (51.4%)	9 (52.9%)	4 (44.4%)	1.00
	<65	18 (48.6%)	8 (47.1%)	5 (55.6%)	
Gender	Male	22 (59.5%)	7 (41.2%)	5 (55.6%)	0.49
	Female	15 (40.5%)	10 (58.8%)	4 (44.4%)	
Histology	L-sarcoma	24 (64.9%)	3 (17.7%)	6 (66.7%)	0.0036
	Non–L–sarcoma	13 (35.1%)	14 (82.3%)	3 (33.3%)	
Primary lesion	Extremities	12 (32.4%)	4 (23.5%)	4 (44.4%)	0.55
	Others	25 (67.6%)	13 (76.5%)	5 (55.6%)	
Resection	Yes	27 (73.0%)	11 (64.7%)	8 (88.9%)	0.45
	No	10 (27.0%)	6 (35.3%)	1 (11.1%)	
ECOG PS	0	5 (13.5%)	3 (17.7%)	1 (11.1%)	0.88
	≥1	32 (86.5%)	14 (82.3%)	8 (88.9%)	
Previous doxorubicin	Yes	23 (62.2%)	12 (70.6%)	7 (77.8%)	0.70
	No	14 (37.8%)	5 (29.4%)	2 (22.2%)	
Previous ifosfamide	Yes	14 (37.8%)	8 (47.1%)	5 (55.6%)	0.59
	No	23 (62.2%)	9 (52.9%)	4 (44.4%)	
No. of previous chemotherapy	0–1	29 (78.4%)	13 (76.5%)	6 (66.7%)	0.77
	≥2	8 (21.6%)	4 (23.5%)	3 (33.3%)	
No. of following chemotherapy	0–1	26 (70.3%)	13 (76.5%)	5 (55.6%)	0.58
	≥2	11 (29.7%)	4 (23.5%)	4 (44.4%)	
Radiotherapy	Yes	16 (43.2%)	8 (47.1%)	6 (66.7%)	0.53
	No	21 (56.8%)	9 (52.9%)	3 (33.3%)	
Carbon-ion radiotherapy	Yes	8 (21.6%)	3 (17.7%)	0 (0.0%)	0.46
	No	29 (78.4%)	14 (82.3%)	9 (100%)	
ALC	Low	27 (73.0%)	10 (58.8%)	8 (88.9%)	0.27
	High	10 (27.0%)	7 (41.2%)	1 (11.1%)	
NLR	Low	19 (51.3%)	9 (52.8%)	4 (44.4%)	1.00
	High	18 (48.7%)	8 (47.2%)	5 (55.6%)	
PLR	Low	17 (45.9%)	9 (52.8%)	3 (33.3%)	0.68
	High	20 (54.1%)	8 (47.2%)	6 (66.7%)	
LMR	Low	17 (45.9%)	7 (41.2%)	5 (55.6%)	0.78
	High	20 (54.1%)	10 (58.8%)	4 (44.4%)	

ECOG PS, Eastern Cooperative Oncology Group performance status. ALC, absolute lymphocyte count. NLR, neutrophil-to-lymphocyte ratio. PLR, platelet-to-lymphocyte ratio. LMR, lymphocyte-to-monocyte ratio.

**Table 2:** Univariate analyses of the baseline factors for OS.

Parameter		HR (95% CI)	*p*-Value
Age	≥65 (vs. <65)	0.61 (0.32–1.19)	0.15
Gender	Male (vs. Female)	1.06 (0.55–2.04)	0.87
Histology	L-sarcoma (vs. non–L–sarcoma)	0.58 (0.30–1.14)	0.11
Primary lesion	Extremities (vs. others)	0.90 (0.45–1.83)	0.78
Resection	Yes (vs. No)	0.57 (0.28–1.15)	0.12
ECOG PS	0 (vs. ≥1)	0.48 (0.15–1.57)	0.22
Previous doxorubicin	Yes (vs. No)	1.11 (0.55–2.25)	0.77
Previous ifosfamide	Yes (vs. No)	1.11 (0.58–2.11)	0.76
No. of previous chemotherapy	0–1 (vs. ≥2)	0.70 (0.34–1.46)	0.34
Radiation	Yes (vs. No)	1.01 (0.53–1.93)	0.97
Carbon-ion radiotherapy	Yes (vs. No)	1.00 (0.41–2.41)	1.00
First drug	Eribulin (vs. others)	0.60 (0.31–1.14)	0.12
	Pazopanib (vs. others)	1.98 (0.99–3.96)	0.053
	Trabectedin (vs. others)	0.93 (0.36–2.40)	0.88
ALC	Low (vs. High)	1.18 (0.58–2.40)	0.64
NLR	Low (vs. High)	0.50 (0.26–0.96)	0.037
PLR	Low (vs. High)	0.52 (0.27–1.02)	0.057
LMR	Low (vs. High)	2.24 (1.17–4.31)	0.015

HR, hazard ratio. 95%CI, 95% confidence interval. ECOG PS, Eastern Cooperative Oncology Group performance status. ALC, absolute lymphocyte count. NLR, neutrophil-to-lymphocyte ratio. PLR, platelet-to-lymphocyte ratio. LMR, lymphocyte-to-monocyte ratio.

**Table 3:** Univariate analyses for OS in the low NLR and the low PLR subgroup.

		Low NLR		Low PLR	
Parameter		HR (95% CI)	*p*-Value	HR (95% CI)	*p*-Value
Age	≥65 (vs. <65)	0.94 (0.35–2.54)	0.91	0.96 (0.31–2.93)	0.94
Gender	Male (vs. Female)	0.49 (0.18–1.34)	0.17	0.70 (0.22–2.17)	0.53
Histology	L-sarcoma (vs. non–L–sarcoma)	0.48 (0.17–1.31)	0.15	0.59 (0.19–1.81)	0.36
Primary lesion	Extremities (vs. others)	0.67 (0.23–1.94)	0.46	0.88 (0.29–2.70)	0.83
Resection	Yes (vs. No)	0.63 (0.22–1.82)	0.39	0.79 (0.22–2.86)	0.71
ECOG PS	0 (vs. ≥1)	1.34 (0.38–4.71)	0.65	1.33 (0.36–4.86)	0.67
Previous doxorubicin	Yes (vs. No)	1.68 (0.58–4.90)	0.34	1.45 (0.47–4.48)	0.52
Previous ifosfamide	Yes (vs. No)	1.54 (0.57–4.15)	0.39	1.01 (0.31–3.30)	0.98
No. of previous chemotherapy	0–1 (vs. ≥2)	0.90 (0.20–4.09)	0.89	0.90 (0.29–2.81)	0.86
Radiotherapy	Yes (vs. No)	0.79 (0.29–2.18)	0.65	0.73 (0.24–2.24)	0.58
Carbon-ion radiotherapy	Yes (vs. No)	1.43 (0.46–4.47)	0.54	1.10 (0.30–4.02)	0.89
First drug	Eribulin (vs. others)	0.40 (0.15–1.08)	0.072	0.32 (0.10–0.98)	0.046
	Pazopanib (vs. others)	5.93 (1.94–18.13)	0.0018	4.71 (1.38–16.04)	0.013
	Trabectedin (vs. others)	0.38 (0.15–2.87)	0.35	0.96 (0.21–4.41)	0.96

HR, hazard ratio. 95%CI, 95% confidence interval. ECOG PS, Eastern Cooperative Oncology Group performance status. ALC, absolute lymphocyte count. NLR, neutrophil-to-lymphocyte ratio. PLR, platelet-to-lymphocyte ratio. LMR, lymphocyte-to-monocyte ratio.

## Data Availability

Data used in the study are available from the corresponding author upon reasonable request.

## Supplementary Materials

The following are available online at https://www.mdpi.com/article/10.3390/jcm10214972/s1, Table S1: Overlapping of the administration of eribulin, pazopanib, and trabectedin. Table S2: Histological subtypes of soft tissue sarcomas. Table S3: Univariate analyses of the baseline factors for PFS. Table S4: Hematological adverse events during the first course of each drug administration. Figure S1: Subgroup analyses of OS among the first administered drugs. Figure S2: Subgroup analyses of PFS among the first administered drugs.
