# Supplementary material for: Does the Use of Peripheral Immune-Related Markers Indicate Whether to Administer Pazopanib, Trabectedin, or Eribulin to Advanced Soft Tissue Sarcoma Patients?
Source: J Clin Med. 2021 Oct 26;10(21):4972. doi: 10.3390/jcm10214972 (PMC8584915; doi:10.3390/jcm10214972)
Supplement: Supplementary file 1 [file jcm-10-04972-s001.zip › jcm-1429121-supplementary.pdf]

Supplementary Table S1. Overlapping of the administration of eribulin, pazopanib, and trabectedin.

Group	Overlapping	*n*
Eribulin	Eribulin only	18 (48.7 %)
	Followed by either pazopanib or trabectedin	12 (32.4 %)
	Followed by both pazopanib and trabectedin	7 (18.9 %)
Pazopanib	Pazopanib only	8 (47.1 %)
	Followed by either eribulin or trabectedin	5 (29.4 %)
	Followed by both eribulin and trabectedin	4 (23.5 %)
Trabectedin	Trabectedin only	1 (11.1 %)
	Followed by either eribulin or pazopanib	4 (44.4 %)
	Followed by both eribulin and pazopanib	4 (44.4 %)

Supplementary Table S2. Histological subtypes of soft tissue sarcomas.

	Eribulin (*n* = 37)	Pazopanib (*n* = 17)	Trabectedin (*n* = 9)
Dedifferentiated liposarcoma	15 (40.5%)	2 (11.8 %)	1 (11.1 %)
Leiomyosarcoma	9 (24.3 %)	1 (5.9 %)	2 (22.2 %)
UPS	7 (18.9 %)	2 (11.8 %)	0 (0.0 %)
Synovial sarcoma	3 (8.1 %)	2 (11.8 %)	1 (11.1 %)
Myxofibrosarcoma	1 (2.7 %)	1 (5.9 %)	1 (11.1 %)
MPNST	1 (2.7 %)	1 (5.9 %)	0 (0.0 %)
Unclassified sarcoma	1 (2.7 %)	2 (11.8 %)	0 (0.0 %)
Angiosarcoma	0 (0.0 %)	2 (11.8 %)	0 (0.0 %)
Malignant solitary fibrous tumor	0 (0.0 %)	2 (11.8 %)	0 (0.0 %)
Adult fibrosarcoma	0 (0.0 %)	1 (5.9 %)	0 (0.0 %)
EMCS	0 (0.0 %)	1 (5.9 %)	0 (0.0 %)
Myxoid liposarcoma	0 (0.0 %)	0 (0.0 %)	3 (33.3 %)
Extraskeletal Osteosarcoma	0 (0.0 %)	0 (0.0 %)	1 (11.1 %)

UPS, Undifferentiated pleomorphic sarcoma. MPNST, Malignant peripheral nerve sheath tumor. EMCS, Extraskeletal myxoid chondrosarcoma.

Supplementary Table S3. Univariate analyses of the baseline factors for PFS.

Parameter		HR (95% CI)	*p*-value
Age	≥ 65 (vs. < 65)	0.62 (0.36–1.09)	0.10
Gender	Male (vs. Female)	0.86 (0.49–1.50)	0.59
Histology	L-sarcoma (vs. non–L–sarcoma)	0.65 (0.37–1.15)	0.14
Primary lesion	Extremities (vs. others)	0.83 (0.45–1.55)	0.56
Resection	Yes (vs. No)	0.99 (0.54–1.82)	0.98
ECOG PS	0 (vs. ≥ 1)	0.87 (0.39–1.94)	0.87
Previous doxorubicin	Yes (vs. No)	1.17 (0.62–2.22)	0.62
Previous ifosfamide	Yes (vs. No)	1.17 (0.67–2.05)	0.58
No. of previous chemotherapy	0–1 (vs. ≥ 2)	0.60 (0.32–1.12)	0.11
Radiotherapy	Yes (vs. No)	1.14 (0.65–1.98)	0.65
Carbon–ion radiotheapy	Yes (vs. No)	0.88 (0.41–1.87)	0.73
First drug	Eribulin (vs. others)	0.61 (0.34–1.07)	0.084
	Pazopanib (vs. others)	1.38 (0.76–2.53)	0.29
	Trabectedin (vs. others)	1.67 (0.75–3.76)	0.21
ALC	Low (vs. High)	1.04 (0.56–1.90)	0.91
NLR	Low (vs. High)	1.06 (0.59–1.89)	0.85
PLR	Low (vs. High)	1.14 (0.64–2.04)	0.65
LMR	Low (vs. High)	1.38 (0.79–2.41)	0.26

HR, hazard ratio. 95%CI, 95% confidence interval. ECOG PS, Eastern Cooperative Oncology Group performance status. ALC, absolute lymphocyte count. NLR, neutrophil–to–lymphocyte ratio. PLR, platelet–to–lymphocyte ratio. LMR, lymphocyte–to–monocyte ratio.

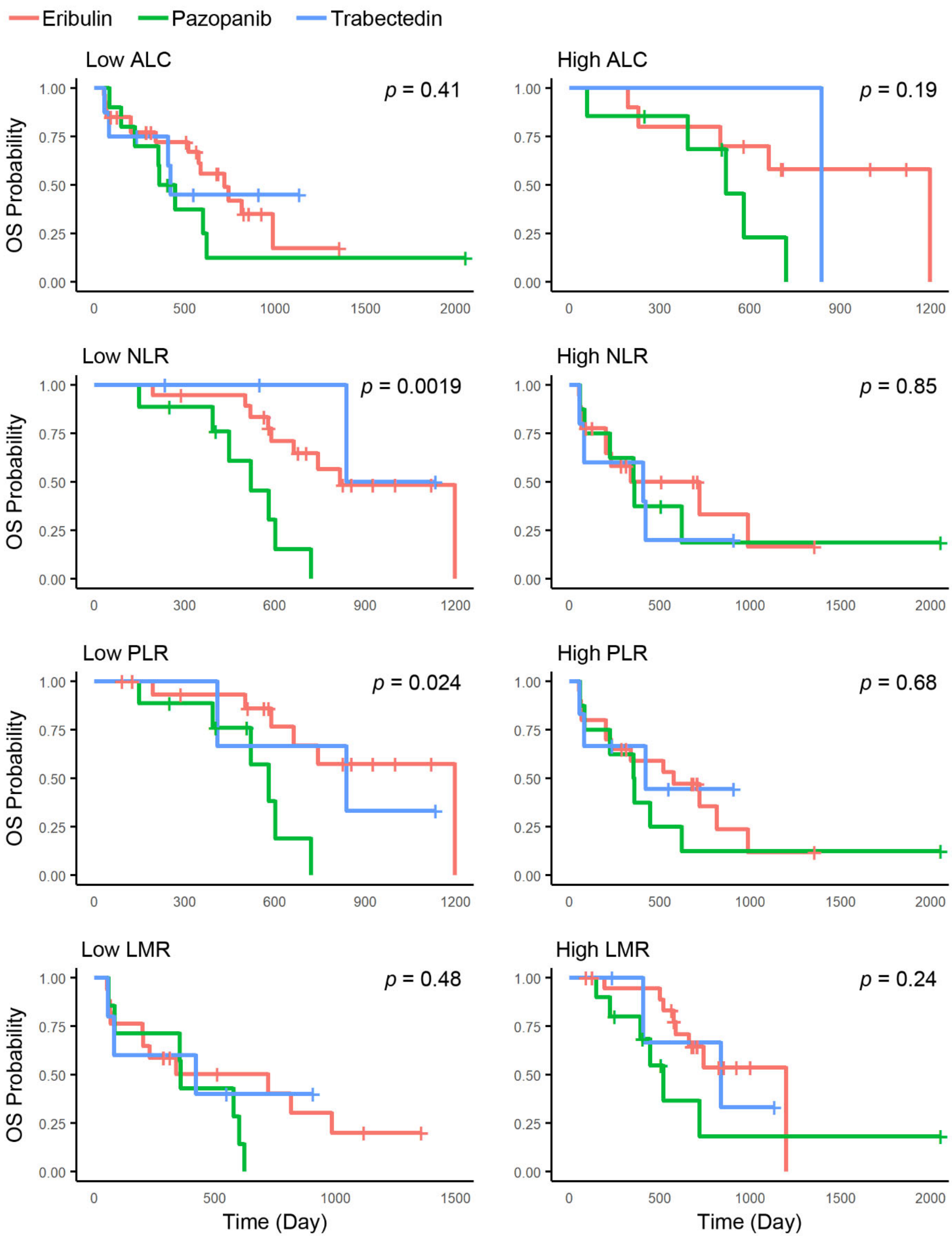

Supplementary Figure S1. Subgroup analyses of OS among the first administered drugs. There were significant differences among the first administered drugs in the low NLR subgroup and the low PLR subgroup. ALC, absolute lymphocyte count. NLR, neutrophil–to–lymphocyte ratio. PLR, platelet–to–lymphocyte ratio. LMR, lymphocyte–to–monocyte ratio.

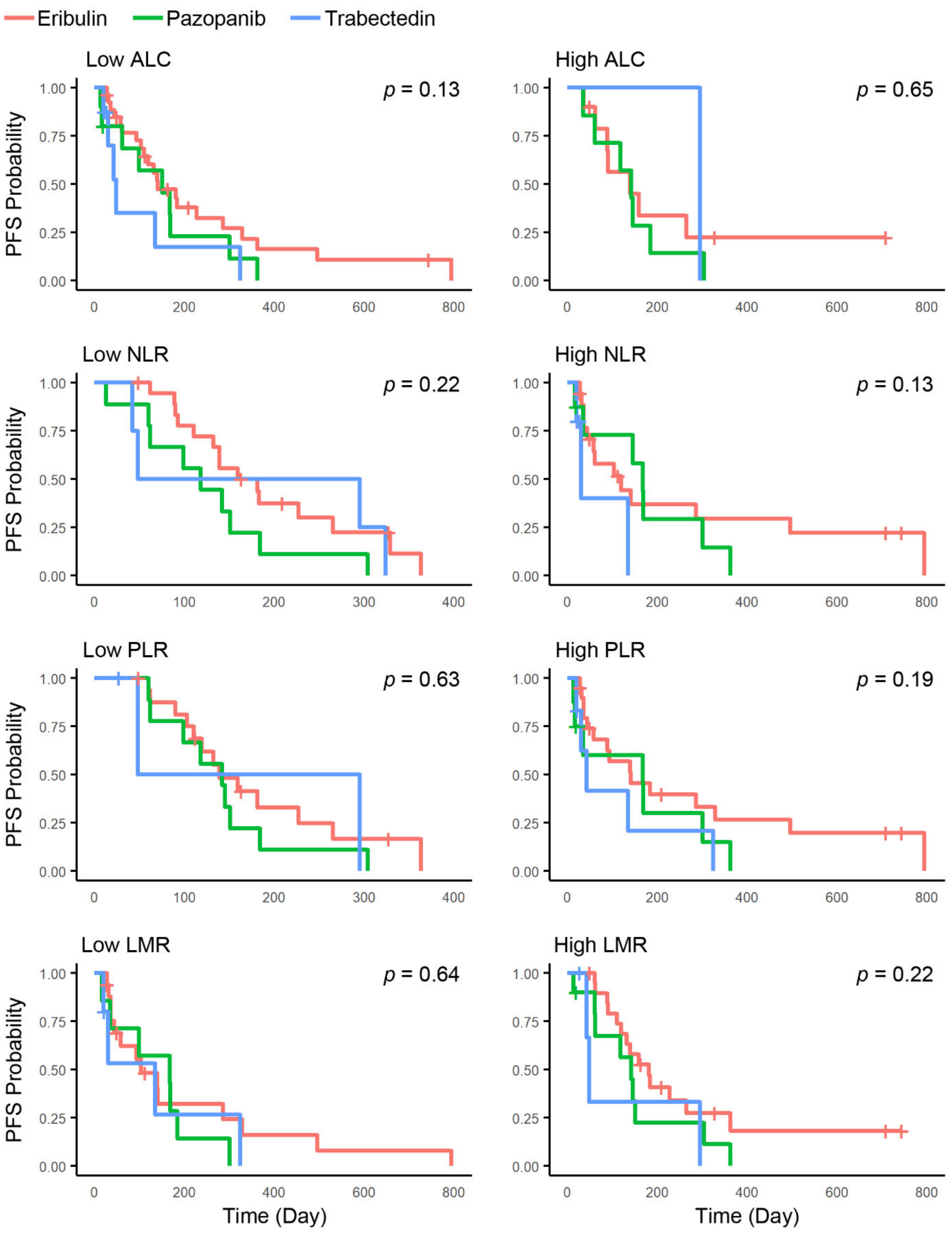

Supplementary Figure S2. Subgroup analyses of PFS among the first administered drugs. There were no significant differences among the first administered drugs in each subgroup. ALC, absolute lymphocyte count. NLR, neutrophil–to–lymphocyte ratio. PLR, platelet–to–lymphocyte ratio. LMR, lymphocyte–to–monocyte ratio.

Supplementary Table S4. Hematological adverse events during the first course of each drug administration.

			ALC			NLR			PLR			LMR		
			**Low**	**High**	***p*-value**	**Low**	**High**	***p*-value**	**Low**	**High**	***p*-value**	**Low**	**High**	***p*-value**
Eribulin	Neutropenia	none	3 (11.1 %)	2 (20.0 %)	0.30	3 (15.8 %)	2 (11.1 %)	0.24	2 (11.8 %)	3 (15.0 %)	0.81	3 (17.6 %)	2 (10.0 %)	0.78
		G1	4 (14.8 %)	2 (20.0 %)		1 (5.3 %)	5 (27.8 %)		2 (11.8 %)	4 (20.0 %)		3 (17.6 %)	3 (15.0 %)	
		G2	6 (22.2 %)	4 (40.0 %)		6 (31.6 %)	4 (22.2 %)		5 (29.4 %)	5 (25.0 %)		5 (29.4 %)	5 (25.0 %)	
		G3	6 (22.2 %)	2 (20.0 %)		3 (15.8 %)	5 (27.8 %)		5 (29.4 %)	3 (15.0 %)		4 (23.5 %)	4 (20.0 %)	
		G4	8 (29.6 %)	0 (0.0 %)		6 (31.6 %)	2 (11.1 %)		3 (17.6 %)	5 (25.0 %)		2 (11.8 %)	6 (30.0 %)	
	Lymphopenia	none	8 (29.6 %)	8 (80.0 %)	0.049	10 (52.6 %)	6 (33.3 %)	0.18	12 (70.6 %)	4 (20.0 %)	0.014	6 (35.3 %)	10 (50.0 %)	0.50
		G1	9 (33.3 %)	2 (20.0 %)		6 (31.6 %)	5 (27.8 %)		2 (11.8 %)	9 (45.0 %)		6 (35.3 %)	5 (25.0 %)	
		G2	6 (22.2 %)	0 (0.0 %)		3 (15.8 %)	3 (16.7 %)		2 (11.8 %)	4 (20.0 %)		2 (11.8 %)	4 (20.0 %)	
		G3	4 (14.8 %)	0 (0.0 %)		0 (0.0 %)	4 (22.2 %)		1 (5.9 %)	3 (15.0 %)		3 (17.6 %)	1 (5.0 %)	
		G4	0 (0.0 %)	0 (0.0 %)		0 (0.0 %)	0 (0.0 %)		0 (0.0 %)	0 (0.0 %)		0 (0.0 %)	0 (0.0 %)	
	Thrombocytopenia	none	24 (88.9 %)	10 (100.0 %)	1.00	17 (89.5 %)	17 (94.4 %)	1.00	14 (82.4 %)	20 (100.0 %)	0.088	17 (100.0 %)	17 (85.0 %)	0.49
		G1	1 (3.7 %)	0 (0.0 %)		1 (5.3 %)	0 (0.0 %)		1 (5.9 %)	0 (0.0 %)		0 (0.0 %)	1 (5.0 %)	
		G2	2 (3.9 %)	0 (0.0 %)		1 (2.6 %)	1 (5.6 %)		2 (6.1 %)	0 (0.0 %)		0 (0.0 %)	2 (10.0 %)	
		G3	0 (0.0 %)	0 (0.0 %)		0 (0.0 %)	0 (0.0 %)		0 (0.0 %)	0 (0.0 %)		0 (0.0 %)	0 (0.0 %)	
	FN	none	24 (88.9 %)	10 (100.0 %)	0.55	19 (100.0 %)	15 (83.3 %)	0.11	16 (94.1 %)	18 (90.0 %)	1.00	15 (88.2 %)	19 (95.0 %)	0.58
		G3	3 (11.1 %)	0 (0.0 %)		0 (0.0 %)	3 (16.7 %)		1 (5.9 %)	2 (10.0 %)		2 (11.8 %)	1 (5.0 %)	
Pazopanib	Neutropenia	none	4 (40.0 %)	5 (71.4 %)	0.55	5 (55.6 %)	4 (50.0 %)	1.00	5 (55.6 %)	4 (50.0 %)	1.00	3 (42.9 %)	6 (60.0 %)	0.81
		G1	4 (40.0 %)	1 (14.3 %)		2 (22.2 %)	3 (37.5 %)		2 (22.2 %)	3 (37.5 %)		3 (42.9 %)	2 (20.0 %)	
		G2	2 (20.0 %)	1 (14.3 %)		2 (22.2 %)	1 (12.5 %)		2 (22.2 %)	1 (12.5 %)		1 (14.3 %)	2 (20.0 %)	
		G3	0 (0.0 %)	0 (0.0 %)		0 (0.0 %)	0 (0.0 %)		0 (0.0 %)	0 (0.0 %)		0 (0.0 %)	0 (0.0 %)	
		G4	0 (0.0 %)	0 (0.0 %)		0 (0.0 %)	0 (0.0 %)		0 (0.0 %)	0 (0.0 %)		0 (0.0 %)	0 (0.0 %)	
	Lymphopenia	none	3 (30.0 %)	5 (71.4 %)	0.49	7 (77.8 %)	1 (12.5 %)	0.032	7 (77.8 %)	1 (12.5 %)	0.032	2 (28.6 %)	6 (60.0 %)	0.062
		G1	3 (30.0 %)	1 (14.3 %)		1 (11.1 %)	3 (37.5 %)		1 (11.1 %)	3 (37.5 %)		3 (42.9 %)	1 (10.0 %)	
		G2	2 (20.0 %)	1 (14.3 %)		1 (11.1 %)	2 (25.0 %)		1 (11.1 %)	2 (25.0 %)		0 (0.0 %)	3 (30.0 %)	
		G3	2 (20.0 %)	0 (0.0 %)		0 (0.0 %)	2 (25.0 %)		0 (0.0 %)	2 (25.0 %)		2 (28.6 %)	0 (0.0 %)	
		G4	0 (0.0 %)	0 (0.0 %)		0 (0.0 %)	0 (0.0 %)		0 (0.0 %)	0 (0.0 %)		0 (0.0 %)	0 (0.0 %)	
	Thrombocytopenia	none	6 (60.0 %)	4 (57.1 %)	1.00	4 (44.4 %)	6 (75.0 %)	0.46	4 (44.4 %)	6 (75.0 %)	0.46	5 (71.4 %)	5 (50.0 %)	0.78
		G1	3 (30.0 %)	3 (42.9 %)		4 (44.4 %)	2 (25.0 %)		4 (44.4 %)	2 (25.0 %)		2 (28.6 %)	4 (40.0 %)	
		G2	0 (0.0 %)	0 (0.0 %)		0 (0.0 %)	0 (0.0 %)		0 (0.0 %)	0 (0.0 %)		0 (0.0 %)	0 (0.0 %)	
		G3	1 (10.0 %)	0 (0.0 %)		1 (11.1 %)	0 (0.0 %)		1 (11.1 %)	0 (0.0 %)		0 (0.0 %)	1 (10.0 %)	
	FN	none	10 (100.0 %)	7 (100.0 %)	1.00	9 (100.0 %)	8 (100.0 %)	1.00	9 (100.0 %)	8 (100.0 %)	1.00	7 (100.0 %)	10 (100.0 %)	1.00
		G3	0 (0.0 %)	0 (0.0 %)		0 (0.0 %)	0 (0.0 %)		0 (0.0 %)	0 (0.0 %)		0 (0.0 %)	0 (0.0 %)	
Trabectedin	Neutropenia	none	5 (62.5 %)	0 (0.0 %)	0.44	1 (25.0 %)	4 (80.0 %)	0.37	1 (33.3 %)	4 (66.7 %)	1.00	4 (80.0 %)	1 (25.0 %)	0.37
		G1	1 (12.5 %)	1 (100.0 %)		1 (25.0 %)	1 (20.0 %)		1 (33.3 %)	1 (16.7 %)		1 (20.0 %)	1 (25.0 %)	
		G2	2 (25.0 %)	0 (0.0 %)		2 (50.0 %)	0 (0.0 %)		1 (33.3 %)	1 (16.7 %)		0 (0.0 %)	2 (50.0 %)	
		G3	0 (0.0 %)	0 (0.0 %)		0 (0.0 %)	0 (0.0 %)		0 (0.0 %)	0 (0.0 %)		0 (0.0 %)	0 (0.0 %)	
		G4	0 (0.0 %)	0 (0.0 %)		0 (0.0 %)	0 (0.0 %)		0 (0.0 %)	0 (0.0 %)		0 (0.0 %)	0 (0.0 %)	
	Lymphopenia	none	1 (12.5 %)	0 (0.0 %)	0.33	1 (25.0 %)	0 (0.0 %)	0.71	0 (0.0 %)	1 (16.7 %)	0.57	1 (20.0 %)	0 (0.0 %)	1.00
		G1	2 (25.0 %)	0 (0.0 %)		1 (25.0 %)	1 (20.0 %)		0 (0.0 %)	2 (33.3 %)		1 (20.0 %)	1 (25.0 %)	
		G2	4 (50.0 %)	0 (0.0 %)		1 (25.0 %)	3 (60.0 %)		2 (66.7 %)	2 (33.3 %)		2 (40.0 %)	2 (50.0 %)	
		G3	0 (0.0 %)	1 (100.0 %)		1 (25.0 %)	0 (0.0 %)		1 (33.3 %)	0 (0.0 %)		0 (0.0 %)	1 (25.0 %)	
		G4	1 (12.5 %)	0 (0.0 %)		0 (0.0 %)	1 (20.0 %)		0 (0.0 %)	1 (16.7 %)		1 (20.0 %)	0 (0.0 %)	
	Thrombocytopenia	none	7 (87.5 %)	0 (0.0 %)	0.22	2 (50.0 %)	5 (100.0 %)	0.17	1 (33.3 %)	6 (100.0 %)	0.083	5 (100.0 %)	2 (50.0 %)	0.17
		G1	1 (12.5 %)	1 (100.0 %)		2 (50.0 %)	0 (0.0 %)		2 (66.7 %)	0 (0.0 %)		0 (0.0 %)	2 (50.0 %)	
		G2	0 (0.0 %)	0 (0.0 %)		0 (0.0 %)	0 (0.0 %)		0 (0.0 %)	0 (0.0 %)		0 (0.0 %)	0 (0.0 %)	
		G3	0 (0.0 %)	0 (0.0 %)		0 (0.0 %)	0 (0.0 %)		0 (0.0 %)	0 (0.0 %)		0 (0.0 %)	0 (0.0 %)	
	FN	none	8 (100.0 %)	1 (100.0 %)	1.00	4 (100.0 %)	5 (100.0 %)	1.00	3 (100.0 %)	6 (100.0 %)	1.00	5 (100.0 %)	4 (100.0 %)	1.00
		G3	0 (0.0 %)	0 (0.0 %)		0 (0.0 %)	0 (0.0 %)		0 (0.0 %)	0 (0.0 %)		0 (0.0 %)	0 (0.0 %)	

ALC, absolute lymphocyte count. NLR, neutrophil–to–lymphocyte ratio. PLR, platelet–to–lymphocyte ratio. LMR, lymphocyte–to–monocyte ratio. FN, febrile neutropenia.
